# Pathogenesis and Function of Interleukin-35 in Rheumatoid Arthritis

**DOI:** 10.3389/fphar.2021.655114

**Published:** 2021-05-13

**Authors:** Pan Lin Xin, Li Fan Jie, Qian Cheng, Du Yi Bin, Cheng Wen Dan

**Affiliations:** ^1^ School of Life Sciences, Anhui Medical University, Hefei, China; ^2^ Department of Orthopedic, Third Affiliated Hospital of Anhui Medical University, Hefei, China; ^3^ Research and Experimental Center of Anhui Medical University, Hefei, China; ^4^ Second Hospital of Anhui Medical University, Hefei, China

**Keywords:** rheumatoid arthritis, IL-35, IL-12 family, treg, Th17, article highlights

## Abstract

It is well known that RA (Rheumatoid arthritis) is an autoimmune disease characterized by multiple and symmetric arthropathy. The main pathological features of RA are synovial hyperplasia, angiogenesis, pannus formation, inflammatory cell infiltration, articular cartilage, bone destruction, and ultimately joint dysfunction, even deformity. IL-35 (Interleukin-35) is a new member of the IL-12 (Interleukin-12) family, which is an immunosuppressive and anti-inflammatory cytokine secreted mainly by Treg (T regulatory cells). There is evidence suggested that IL-35 can attenuate the progression of RA through influencing the immune and pathological process. It suggests that IL-35 played an important role in the pathogenesis of RA, and can be used as a potential target for the future treatment of RA. This review summarizes the recent advances of IL-35 in the pathological roles and the therapeutic potential roles in RA.

## Highlights

1. IL-35 is an inhibitory cytokine that closely related to the occurrence and development of RA.
2. IL-35 can induce the proliferation of Treg and inhibit the differentiation of Th17.
3. IL-35 can relieve the symptoms of RA greatly by inhibiting the proliferation of FLS, angiogenesis, and bone destruction.

## Introduction

RA is an autoimmune disease characterized by multiple and symmetric arthropathy (El et al., 2007). It is associated with a significant morbidity and disability rate. Recent epidemiological investigations reveals that the total incidence of RA is 1–2% in the world. If untreated, the 2–3 years disability rate of RA can be as high as 70% (Jutley et al., 2015). It seriously affects the living quality. The main pathological features of RA are synovial hyperplasia, angiogenesis, pannus formation, inflammatory cell infiltration, articular cartilage, bone destruction, and ultimately joint dysfunction, even deformity (Turk et al., 2014; Elshabrawy et al., 2015). Although the specific pathogenesis of RA is very complicated, it is considered that the incidence of RA is related to the IL-12 family (Guan et al., 2017).

IL-12 family is composed of IL-12, IL-23, IL-27, IL-35. It helps to regulate the immune system against from infectious diseases, tumors, autoimmune disease (Gee et al., 2009). As a new member of the IL-12 family, IL-35 is first found in the supernatant of B lymphoblastoid cells infected by Epstein-Barr virus in 2007 (Collison et al., 2007). IL-35 is an immunosuppressive and anti-inflammatory cytokine which mainly secrets by Tregs (Bobryshev et al., 2015). It can induce the proliferation of Tregs and inhibit the differentiation of Th17 (T Helper cell 17) cells (Wang et al., 2014; Behzadi et al., 2016). IL-35 is closely related to the occurrence of infections, inflammation, tumors, autoimmune diseases, such as primary biliary cirrhosis, Crohn’s disease, systemic sclerosis, and RA (Kong et al., 2016; Zou et al., 2017; Teymouri et al., 2018; Zhao et al., 2020; Zhu et al., 2020).

Collectively, these data suggests that exploring the biological functions and mechanisms of IL-35 may play a pivotal role in the pathogenesis of RA. The present review provides a brief overview of the IL-35, discusses the IL-35 involves in the pathogenesis of RA, and the therapeutic potential of IL-35 in RA.

### Overview of IL-35

**FIGURE 1:**
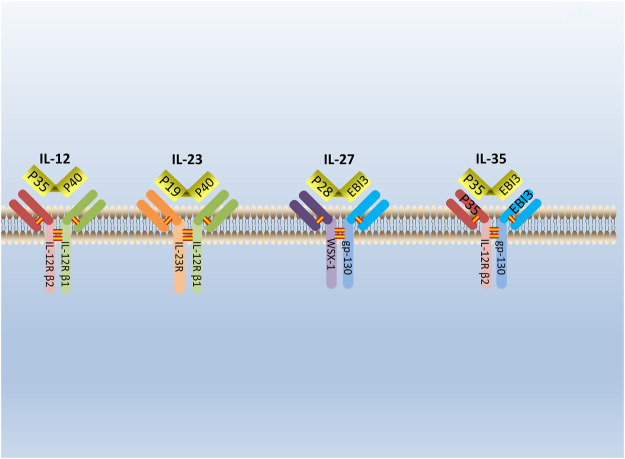
The structure of IL-12 family. IL-12 family is composed of A chains (p19, p28, or p35) and B chains (p40 or Ebi3). P40 can be paired with p35 or p19 to form IL-12 or IL-23. Respectively, while Ebi3 can pair with p28 or p35 to form IL-27 or IL-35. IL-12 receptor is composed of IL-12R β2 and IL-12R β1. IL-23 receptor is composed of IL-12R β1 and IL-23R. IL-27 receptor is composed of WSX-1and gp-130. IL-35 receptor is composed of IL-12R β2 and gp-130.

IL-35 is a new type of cytokine discovered simultaneously by Collison et al. and Niedbala et al., in 2007 (Collison et al., 2007; Niedbala et al., 2007). It is combined with IL-12, IL-23, and IL-27 to form the IL-12 family (Collison and Vignali, 2008). IL-35 is constituted by A chain (p35/IL-12a) and B chain (EBI3/IL-27b) (Su et al., 2018). The glycoprotein encoded by the p35 gene is homologous with IL-6 and G-CSF (granulocyte colony-stimulating factor) and determined the specificity of cytokines. The EBI3 (Epstein-Barr virus-induced gene 3) gene encodes a glycoprotein with a relative molecular weight of 34,000, and 27% of the amino acid sequences are homologous to IL-12 and p40 subunits that belong to the hematopoietic cytokine receptor family (Chehboun et al., 2017). The p35 and EBI3 gene are located on separated chromosomes 3p12-q13.2 and 19p13.3, respectively, in humans; chromosomes 6 and 17qD in the mouse (Devergne et al., 1996). (Figure 1) Moreover, subunits of the IL-35 receptor are shared among IL-12 family members. IL-35 receptors are consisted of IL12Rb2 (IL-12 receptor component) and gp130 (glycoprotein130) (IL-27 receptor component) (Collison et al., 2012).

IL-35 is directly secreted by regulatory T cells, including thymic Tregs and peripheral Tregs (Pflanz et al., 2002). Evidence shows that CD4^+^CD25^+^Foxp3^+^Tregs can secrete IL-35 in both humans and mice (Kaser et al., 2012), and a class of iTreg (induced-Treg) called iTr35 (IL-35 induced Treg) can also secrete IL-35 (Collison et al., 2010). Besides, a series of non-immune cells including tumor cells can also secrete IL-35 (Long et al., 2013; Wang et al., 2013). In general, IL-35 is inducible in Tregs, Bregs, immature dendritic cells, vascular endothelial cells, epithelial cells, and smooth muscle cells in the human body (Shen et al., 2014; Dambuza et al., 2017; Haller et al., 2017; Mohd Jaya et al., 2019). Two subunits of IL-35 have a high expression level in mice, and the p35 subunit is revelated in blood, liver, thymus, and bone marrow (Li et al., 2012). Besides, IL-35 is found in patient’s serum with RA (Li et al., 2019a), and IL-35 mRNA expression in RA patients is obviously lower (Zhang et al., 2018). It means that the secretion of IL-35 is difference in different tissue. The expression level change of IL-35 in RA patients suggests that it may participate in the pathogenesis of RA.

### IL-35 Participates in the Pathogenesis of RA

**FIGURE 2:**
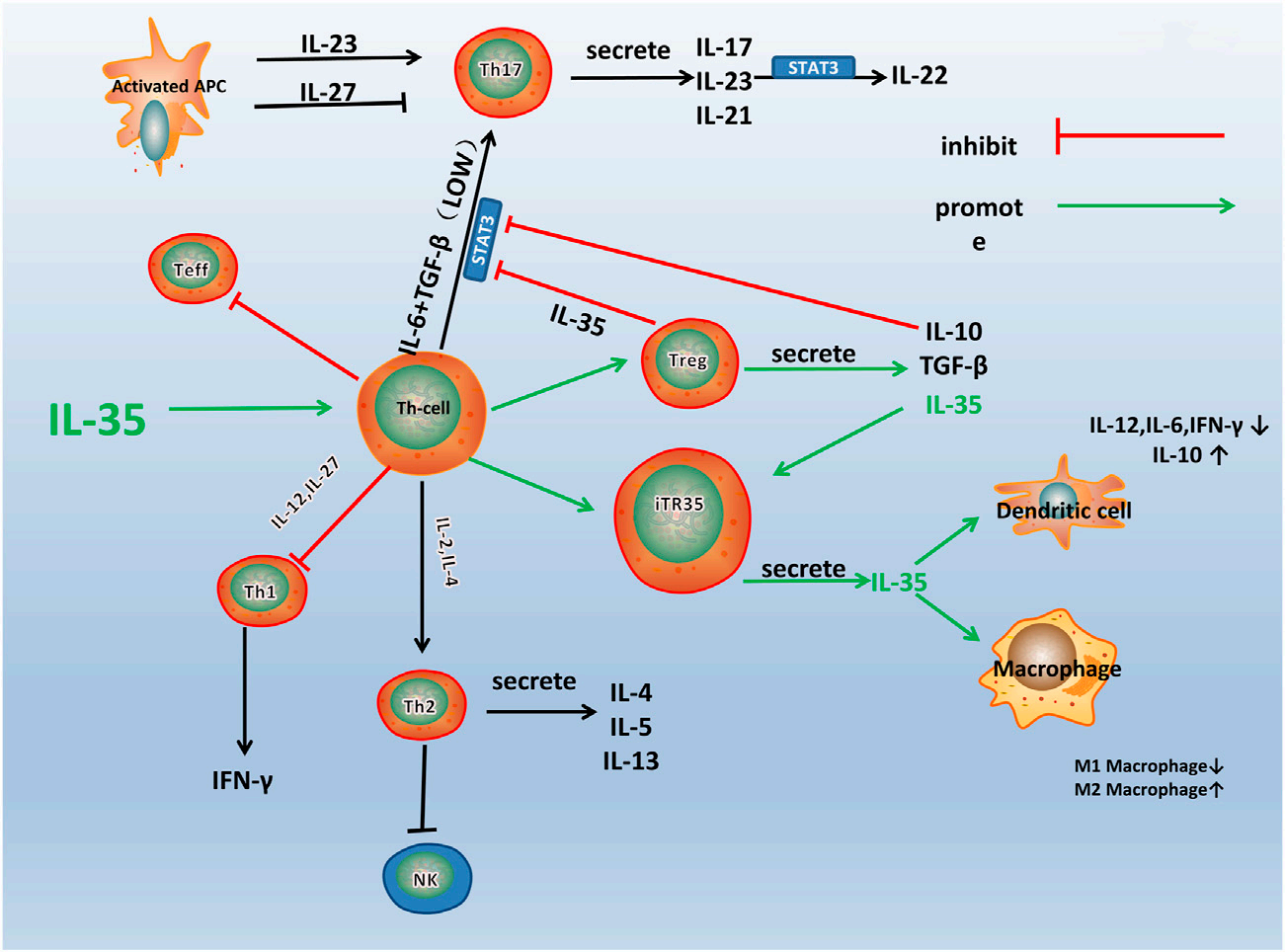
A schematic model for the immune pathway of IL-35 in RA. Th1, Th2, Th17, and Treg are all differentiated from the Th Cells, which can differentiate into different cell subsets under the influence of different cytokines and transcription factors. As shown in the figure, IL-35 stimulates the differentiation of Th cells into Treg and iTR35. IL-10 and TGF-β are secreted by Treg, and IL-35 inhibits the differentiation of Th cells into other helper T cells, especially Th17. IL-35 also acts on DCs and macrophages to inhibit inflammation.

IL-35 participates in various immune-related diseases, such as primary biliary cirrhosis, Crohn’s disease, and RA (Remmers et al., 2010; Bettini et al., 2012; Bossini-Castillo et al., 2012; Zhao et al., 2020). The influence of IL-35 in the pathogenesis of RA is closely related to the immune dysfunction of these immune cells and pathological process (Figure 2). IL-35 promotes the proliferation of Tregs and inhibited the differentiation of Th17 cells (Liu S. et al., 2019). A variety of immune cells including Tregs and Th17 cells infiltrates into the joint and are crucial for synovial inflammation and joint destruction (Wang and Lei, 2021). Therefore, IL-35 playss an important role in the pathological process of RA by maintaining the balance of Tregs and Th17 cells.

### IL-35 Influences the Balance of Immune Cells

IL-35 is directly secreted by Tregs. The variation of the number of Tregs was accompanied by a defect in its function (Lin et al., 2014; Li et al., 2019), and the number of Tregs was found decreased in peripheral blood with active RA patients (Wang et al., 2012). Coincidentally, two chains that constituted IL-35, mRNA of EBI3 and IL-12p35 were found continuously co-expressed at a high expression level in Tregs (Collison et al., 2007). It meaned that the occurrence of RA was closely related to the number and function of Tregs, and IL-35 played an important role in exerting the maximal immunosuppressive function of Tregs. Tregs exerted immunostimulatory activity mainly by EBI3, which could induce macrophages to synthesize macrophage inflammatory protein-1 and further recruit B and T lymphocytes to inflammatory sites (Liu et al., 2012). Further research showed that IL-35 could promote the proliferation of Tregs (Wang and Lei, 2021). A class of Treg called iTR35 cells presented high reactivity and strong inhibition of inflammation by transforming initial T cells into a new type of Foxp3^−^Treg (Forkhead Box P3) (Collison et al., 2010), but it was irrelevant to IL-10, TGF-β (transforming growth factor-β), and other Treg-related immune molecules (Hori et al., 2017). More importantly, iTR35 cells were more stable *in vivo* and had a more durable immune tolerance effect compared with Tregs (Song and Ma, 2016). In addition to the stimulation of recombinant IL-35, it was confirmed that human Tregs derived from umbilical cord blood or adult peripheral blood mononuclear cells could also induce initial T cells to transform into iTR35 cells by secreting IL-35 (Chaturvedi et al., 2011). Taken together, IL-35 might have a significant impact on RA by promoting the differentiation of Tregs.

IL-35 also inhibited the differentiation of Th17 cells. There was a balance between Th17 cells and Tregs under different cell induction conditions, while high concentrations of TGF-β made the balance come to the Tregs (Williams et al., 2013). It was found that *in vitro* derived EBI3^−/−^ Th17 cells could produce a significantly higher expression level of IL-17 and IL-22. Spleen cells from EBI3^−/−^ mice immunized with monocytogenes could also produce elevated expression levels of IL-17 and IL-22 (Yang et al., 2008). A study suggested that IL-35 could directly suppress IL-17 expression and Th17 differentiation (Veldhoen et al., 2006). Furthermore, it was found that the differentiation of Th17 cells was accelerated by TGF-β, while IL-35 could significantly up-regulate the expression level of IFN-γ (Interferon-γ) to inhibit the phosphorylation of Smad-3, a downstream effector of TGF-β receptor, and IL-35 could prevent the binding of TGF-β and its receptor to block the differentiation of Th17 cells (Park et al., 2005). Moreover, there was research that found peripheral blood mononuclear cells which stimulated with IL-35, could inhibit the differentiation and the function of Th17 cells by reducing the relative expression of ROR γt (Nuclear receptor γt) (Xie et al., 2020). Taking all the studies into account, IL-35 could suppress the activity of Th17 cells and play a negative role in the pathogenesis of RA.

### IL-35 Influenced the Pathological Features of RA

The main pathological features of RA are synovial hyperplasia, angiogenesis, pannus formation, inflammatory cell infiltration, articular cartilage, bone destruction, and ultimately joint dysfunction. IL-35 is indicated to participate in the pathological process of RA through several reports.

RA pathogenesis is highly correlated with FLS (Fibroblast-like synoviocytes) which release proteolytic enzymes produced by inflammatory cytokines, such as MMPs (matrix metalloproteinase) (Dinesh and Rasool, 2018). Pro-inflammatory cytokines such as IL-6, IL-8, TNF-α, IL-1β, which causing activation of FLS led to synovial inflammations. IL-35 can restrain the process (Afzali et al., 2007). In a previous experiment, IL-35 inhibits the proliferation and cell cycle progression in FLS which is harvested from CIA mice (Collagen-induced model of RA mice) in a dose-dependent manner. This consequence is accompanied by the downregulation of cyclin D1 (Li et al., 2016a). It indicates that IL-35 inhibits the proliferation of FLS and promoted the apoptosis of FLS directly.

Synovium hyperplasia in RA is accompanied by angiogenesis (Ally et al., 2015) and is a key process in the development of RA. It suggests that RA can be considered as a type of vascular disease, and foster pannus formation, persistent leukocyte infiltration, lining layer hyperplasia, which conclusively lead to cartilage and bone destruction (Szekanecz et al., 2010). Research shows angiogenesis is promoted by VEGF (vascular endothelial growth factor), which is critical in the pannus formation in synovial tissues and plays an important role in RA (Kim et al., 2015). IL-35 is found to downregulate the expression level of VEGF and its receptors in the CIA mice, which shows that IL-35 might influence the pathological procedure of RA (Wu et al., 2016a). The probable mechanisms relied on inhibiting VEGF and its receptors. Except for VEGF, IL-35 can also affect the EC specific factors Ang1, Ang2 (angiopoietin-1) in pathological vascular development. IL-35 is ECs (human umbilical endothelial cells) and RA synovial tissue explants (Jiang et al., 2016a). Thus, this evidence suggests that IL-35 may play a role in antagonizing angiogenesis in the pathological procedure of angiogenesis and pannus formation.

Bone destruction is also one of the most prominent features of RA pathogenesis. It is generally known that the formation and differentiation of osteoclasts rely on the activation of the RANKL receptor (nuclear factor κB ligand) (Tanaka et al., 2018). Previous research suggests that RANKL have a trend of up-regulation in the RA synovial tissues (Gravallese et al., 2000; Liu et al., 2018). Li Y et al. investigates that IL-35 can inhibit the development of RA in CIA mice by a decrease in the expression level of RANKL and an increase in the expression level of OPG (osteoclastogenesis inhibitory factor) (Li et al., 2016a). Additionally, IL-35 is found synergistically inducing osteoclast formation with RANKL (Kamiya et al., 2020). It means IL-35 may play both destructive and protective roles in the formation of osteoclasts. Besides, IL-35 can also inhibit the secretion of MMPs in chondrocytes and synovial fibroblasts and enhance the activities of aggrecanases and collagenase to promote the degradation of cartilage proteoglycan and collagen and heighten the destruction of osteoclasts (Shui et al., 2017; Liu X. et al., 2019; Sun et al., 2019). It means IL-35 do contribution to prevent cartilage matrix and bone destruction. To sum up, IL-35 participates in the pathogenesis of RA, and it has a benefit of teasing out the regulatory mechanism of IL-35 in RA.

### The Regulatory Mechanism of IL- 35 in RA

IL-35 is acknowledged as involved in the pathogenesis of RA by regulating the expression level of pro-inflammatory cytokines (Chen et al., 2019). Its specific regulatory mechanism in RA has been extensively studied in recent years. IL-35 is considered to activate several signal transduction pathways, including JAK-STAT (Janus Kinase-signal transducer and activator of transcription), Ang2-Tie2 (TEK Tyrosine kinase, Endothelial), and Wnt-β-catenin signaling pathways which regulated the procedure of RA.

**FIGURE 3:**
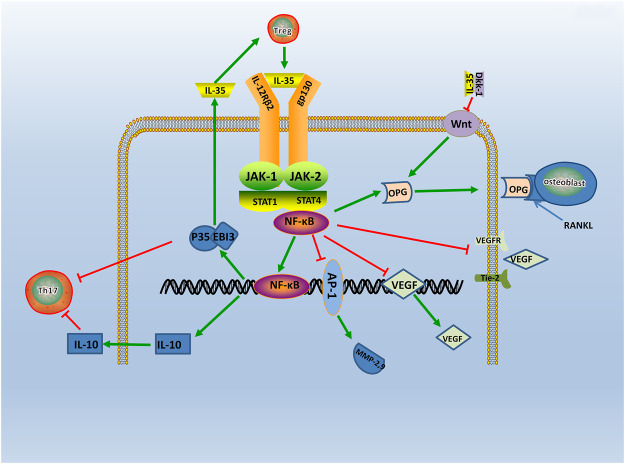
The regulatory mechanism of IL- 35 in RA. IL-35 is secreted by Treg via JAK/STAT signaling pathway. Production of IL-35 and IL-10 triggers Th17 differentiation. In turn, IL-35 which produced by Treg cells, enhances IL-35 expression. Moreover, IL-35 also inhibits Ang2/Tie2 via NF-κB, and with the suppression of VEGF, VEGFR, Tie2, it causes angiogenesis. IL-35 also stimulates OPG secretion and competitively binded RANKL with OPG by inhibiting Wnt/β-catenin signaling pathway, and it inhibits bone destruction in the course of RA. Thus, IL-35 acts as an anti-inflammatory factor participating in RA via various pathways.

Evidence points that the signaling pathway of RA may relate to the JAK-STAT family (Veale et al., 2017). The signaling molecules of the JAK-STAT signaling pathway includes the JAK2, Tyk2, and STAT family (Thierfelder et al., 1996). A study find IL-35 can activate the JAK1 signal transducer and activator of the STAT pathway *in vitro* (Dong et al., 2020). Specifically, two receptors of IL-35, gp130, and IL-12Rβ2, respectively, activates JAK2 and Tyk2 through phosphorylation. The heterodimers or homologous dimers composed of gp130 and IL-12Rβ2 emits signals transferring into the nucleus with activation of the STAT family, then regulated cytokine-specific gene expression and exerting biological effects (Parham et al., 2002). Study find IL-35 increases the expression level of p-STAT1 in FLS, but do not affect the expression level of p-STAT3 and p-STAT5 (Wu et al., 2018). Additionally, JAK inhibitors Tofacitinib is used in RA patients and find that serum expression level of IL-35 is significantly increased (Li et al., 2019). The above results shows that IL-35 can act through the JAK-STAT signaling pathway. Moreover, emerging evidence shows that the phosphorylated (p)-STAT3 is downregulated, and p-STAT1/4 is upregulated in IL-35 overexpressed cells (Cai et al., 2021). These results indicates that IL-35 may impact inflammation through STAT1/4. While the structures and signal transduction of the IL-35 are similar to the other member of IL-12 family, the biological activities are quite opposite (Tait et al., 2019). Therefore, IL-35 may have other unique receptors, and the detailed mechanism needs further study (Figure 3). Thus, it is considered that the signal transduction of IL-35 in the regulation mechanism of RA is closely related to the JAK2, Tyk2, and STAT family.

Evidence shows that the Ang-Tie2 signaling pathway mediates the pathological process of RA (Kayakabe et al., 2012). The role of the Ang-Tie2 signaling pathway is mainly angiogenesis (Vanderborght et al., 2020). In an earlier study, it is observed that constitutive expression of Ang-1 and Ang-2 in late-stage RA synovial fibroblasts (Scott et al., 2002). Recent study shows that Ang-1 and Ang-2 are found to enhance the expression level of pro-inflammatory cytokines in macrophages from donors with RA (Kabala et al., 2020), and evidence shows Tie2 significantly inhibit angiogenesis by neutralizing the Ang2 receptor (Saber et al., 2011). Meanwhile, IL-35 inhibits Ang2 secretion and antagonized the proangiogenic effects of exogenous Ang2 in HUVECs(Jiang et al., 2016b). These results suggests that IL-35 restrained RA angiogenesis and inflammation by disrupting the Ang2-Tie2 signaling pathway.

In addition, studies have shown that the Wnt-β-catenin signaling pathway is also involved in the pathogenesis of RA. The Wnt proteins are glycoproteins which lead to joint formation (Miao et al., 2013), and the Wnt-β-catenin signaling pathway influences the subchondral bone remodeling (Kovacs et al., 2019). Evidence shows Wnt signaling is up-regulated in RA FLS (Sen et al., 2002). The Wnt genes are revealed significant up-regulation in RA patients synovium, and are present in articular cartilage, bone, and synovium of RA patients (Nakamura et al., 2005). Besides, the expression level of Wnt inducible signaling pathway proteins is also found significantly elevated in RA FLS (Cheon et al., 2004). A study shows that the serum expression level of Dkk-1 (Dickkopf-1), an inhibitor of the Wnt-β-catenin signaling pathway, is significantly higher in patients with RA (Wang et al., 2011). It suggests that the Wnt-β-catenin signaling pathway plays a key role in bone resorption and joint destruction during RA development. Meanwhile, it shows that IL-35 activates osteoblastic differentiation via the Wnt-β-catenin signaling pathway in RA, and this result can be resulted by pairing of IL-35 and Dkk-1 (Li et al., 2019). As support, IL-35 is found dose-dependently inhibited the expression level of RANKL and increased the expression level of OPG in cultured FLS, which mainly plays a part through the Wnt-β-catenin signaling pathway (Li et al., 2016b). Therefore, IL-35 may influence the bone resorption and joint destruction in RA by the Wnt-β-catenin signaling pathway.

### The Therapeutic Potential Role of IL-35 in RA

As a potential target, IL-35 is more considered as a monitoring indicator when exploring the pathogenesis of RA (Li et al., 2019; Wojdasiewicz et al., 2019)or predicting the efficacy of certain drugs (Li et al., 2019). Several reports shows the potential of IL-35 in the therapy of RA.

Study shows that IL-35 is secreted in the joint tissue homogenate of CIA mice, and inhibits the inflammatory response of macrophages (Wang and Zhong, 2015). Evidence shows a decreasing arthritis index, and promoting apoptosis in CIA mice, which is injected with IL-35 intraperitoneally (Li et al., 2016b). Moreover, by upregulating the secretion of IL-35, it is found that IL-35 reduces antigen-specific local inflammation and impaired disease development of RA in CIA mice (Yin et al., 2016; Koga et al., 2021). Besides, the antagonist of IL-35 is injected into the joint space and exerted the highest anti-inflammatory effects (Abdi et al., 2018). IL-35 is also found dose-dependently inhibiting the expression level of RANKL and increasing the expression of OPG (Li et al., 2016c). Moreover, it downregulates the expression level of VEGF and its receptors in CIA mice, which is important for angiogenesis (Wu et al., 2016b). While another study shows similar consequences by using a murine Matrigel plugs model (Jiang et al., 2016c). Taking all these studies into account, IL-35 can inhibit the development of RA effectively in mice. But, It also shows a statistically significant increase in clinical scores of CIA after receiving an injection of two distinct plasmids encoding IL-35 gene 3 and 18 days in CIA mice (Thiolat et al., 2014). It means that IL-35 may be elevated in early RA and play a negative role.

Notably, secretion of IL-35 is investigated in synvium from RA patients, and EBI-3/p35 transcripts are expressed (Kam et al., 2018a). A study shows that IL-35 suppressed T cell activation during the peripheral immune responses of RA patients (Li et al., 2019b), and evidence shows IL-35 has a lower expression level in peripheral blood of RA patients than controls (Sun et al., 2015). Several studies have confirmed this result (Ning et al., 2015; Xie et al., 2021). It suggests that IL-35 correlates with the pathogenesis of RA *in vivo*. The serum expression level of IL-35 and the number of Tregs are found to decrease significantly in peripheral blood harvested from patients with RA (Nakano et al., 2015a). Further studies show that low expression level of serum IL-35 correlates with high ESR and DAS28-ESR (DAS28 erythrocyte sedimentation rate) in RA patients (Li et al., 2019c). Thus, the expression level of IL-35 is correlated with the severity of RA. The study evaluates the effect of IL-35 on human osteoclastogenesis, and find it significantly decreased the expression level of RANK mRNA in monocytes in a dose-dependent manner (Yago et al., 2018). Besides, IL-35 shows an inhibiting effect on endothelial cell migration, adhesion, and tube formation in HUVEC (Jiang et al., 2016d). It indicates that IL-35 may have therapeutic potential for RA by inhibiting osteoclastogenesis and angiogenesis. Interestingly, a study shows the expression level of serum IL-35 is significantly higher in patients with treatment naïve early RA compared to controls, and significantly decreased after treatment (Jiang et al., 2016d). It may provide us an opportunity to control early RA by regulating the expression level of IL-35 in the human body. Thus, it is imperative to find a way for IL-35 to play a greater role in RA treatment.

**TABLE 1:** IL-12 family in RA.

Cytokines	Producing cells	Function	References
IL-12	monocytes, macrophages,DCs,B cells	Th1 differentiation; Th2 inhibition; IFN-γ↑	Wu et al. 2016
IL-23	monocytes, macrophages,DCs,B cells, endothelial cells	Th17 differentiationIL-17↑ ; Proliferation of CD4^+^ memory T cells	Nakano et al. 2015b
IL-27	macrophages,DCs,B cells	Inhibition of immune system responses; limit the inflammatory	Behzadi et al. 2016
IL-35	Tregs, breg, endothelial cells; monocytes	Treg and breg differentiation; iTR35 differentiation; Th17 inhibition; teff inhibition; IL-35 and IL-10 ↑; M1macrophages↓; M2macrophages↑	Choi et al. 2015

**TABLE 2:** The targets of IL-35 in RA.

Modulators	IL-35	Target	Model	References
	IL-35	IFN-γ	RA serum	Niedbala et al. 2007
Breg	IL-35	IL-10	PBMCs	Wang et al. 2014
iTR35	IL-35	IL-35	Mice	Collison et al. 2010
	IL-35	TNF-α	FLS	Wu et al. 2016c
Endothelial cells	IL-35	Ang2	Huvec	Jiang et al. 2016a
	IL-35	RANKL	FLS	Li et al. 2016a
	IL-35	OPG	CIA mice	Li et al. 2016d
	IL-35	P-STAT1	FLS	Wu et al. 2018
	IL-35	VEGF	FLS	Wu et al. 2018
Macrophages	IL-35	CCR-7	PBMCs	Peng et al. 2019
CD39^+^CD4^+^T cells	IL-35	Foxp3	Arthritis mice	Filkova et al. 2015
Th-17	IL-35	IL-17	Mice spleen cells	Kam et al. 2018b

**TABLE 3:** Expression of IL-35 in RA.

IL-35	Sample	Expression change	References
IL-35	RA synovium		Kam et al. 2018b
IL-35	Lyme arthritis/CIA mice tissue	+	Kuo et al. 2011
IL-35	Active RA serum		Kam et al. 2018b
IL-35	RA SF	+	Filkova et al. 2015
IL-35	Cultured FLS	+	Wu et al. 2018
IL-35	Normal human serum		Kam et al. 2018b

+:up-regulation, —:down-regulation.

## Future Perspectives

**FIGURE 4:**
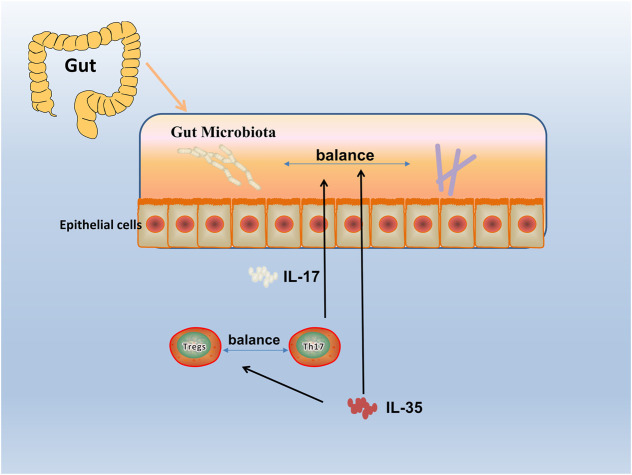
Gut microbiota in RA. The human gut microbiota has a critical role in the pathogenesis of RA. By an interdependent functional relationship, the composition and functions of the gut microbiota plays a role in regulating the Th17/Treg cells balance and affected the host immune responses. Bacteroides fragilis as a normal symbiotic bacterium in the human gut, it mediates the conversion of CD4^+^ T cells to interleukin (IL)-10 through a specific molecule, polysaccharide a (PSA), to produce Foxp3+ Treg cells. While Segmented filamentous bacterium (SFB), commensal, is found inducing and activating of Th17 cells in the lamina propria, which subsequently secreted pro-inflammatory cytokine (IL-17), and enhanced mucosal immune responses of the host.

Since the discovery of IL-35, great progress has been made in exploring the expression, regulation, and function of IL-35 in autoimmune diseases. With the deepening research, the relationship between immune-related diseases such as RA and gut microbiota has been gradually discovered (Wu et al., 2016). Studies have shown that gut microbiota protected humans and mice away from RA by regulating intestinal and parenteral regulation. The intestinal immune response depends on the composition of microorganisms, which produces a series of enzymes, chemicals, hormones, vitamins, etc., that could interact with the metabolism of the host. When RA-related bacteria appeares in large quantities, the imbalance of gut microbiota reduces the growth of probiotics, thus inducing the incidence of RA (Maeda and Takeda, 2017; Balakrishnan et al., 2019), and the change of gut microbiota may occur before the onset of RA (Jeong et al., 2019). Evidence reveales that IL-12 can induce microbiota-driven chronic intestinal inflammation in inflammatory bowel disease (Eftychi et al., 2019a). Study shows the potential therapeutic utility of IL-12 about the gut microbiota, which in a murine model of chronic intestinal inflammation systemically treated with antibodies to IL-12 (Duchmann et al., 1996), and IL-23 is found to regulated gut microbiota to trigger severe intestinal inflammation in animal models (Kullberg et al., 2006). Faecal samples are collected to analyze the microbiota community profiles using next-generation sequencing (Rehaume et al., 2019) and find an abundance of specific strains changed with disease severity in mice treated with anti-IL-23 (Manasson et al., 2020). Furthermore, evidence shows it can affect the composition of the gut microbiota by using IL-27 and antibody against IL-27 in mice (Lavoie et al., 2020). (Figure 4) To sum up, the research progress on gut microbiota could provides a choice for us to regulate intestinal and extraintestinal immune responses, and the IL-12 family including IL-12, IL-23, IL-27 could influence the progression of autoimmune diseases. Therefore, as a member of the IL-12 family, it is worth exploring whether IL-35 could also influence autoimmune diseases such as RA by affecting gut microbiota balance.

Meanwhile, growing gene-editing technology is offering help to treat various diseases through gene knockout or gene loss. For example, with CRISPR-Cas9 targeting the BCL11A erythroid-specific enhancer, γ-globin expression and fetal hemoglobin in erythroid cells is promoted in Transfusion-dependent β-thalassemia and sickle cell disease (Frangoul et al., 2021), and it is reported that CRISPR gene editing is used for cancer immunotherapy by CRISPR-Cas9 editing to engineer T cells in the treatment of refractory cancer (Stadtmauer et al., 2020). Generally, for precisely manipulating cellular DNA sequences and altering cell fates and organism traits, the technique of using CRISPR will soon be in the clinic for several diseases (Doudna, 2020). Moreover, CRISPR is already participated in the clinical application of autoimmune diseases (Safari et al., 2018). Study shows that M1/M2 monocytes imbalance strongly contributes to pathogenesis of RA (Fukui et al., 2017). CRISPR/Cas9 is used to knock out GRK2 gene, which has a capacity in regulating M1/M2 monocytes imbalance, and shows a potential for treating patients with RA by downregulating M1/M2 ratio (Tardito et al., 2019). Another study shows that miR-155 gene-knockdown macrophage cells are impaired in producing proinflammatory cytokines, but increased in osteoclastogenesis (Jing et al., 2015). A CRISPR/Cas9 system which target on IFN-γ gene is designed in inflammatory bowel disease mice models. It is observed that the IFN-γ is deficient, and the damage to the intestinal epithelial cells are prevented (Eftychi et al., 2019b). Taken together, CRISPR/Cas9 shows a potiential in changing the balance of immune cells or the secretion of inflammatory cytokines. Therefore, modulating the expression level of IL-35 through gene-editing technology may become a new therapy for RA.

## Conclusion

In conclusion, as a new member of the IL-12 family, IL-35 is a heterodimeric protein composed of two subunits, EBI3, and IL-12p35. IL-35 is an inhibitory cytokine secreted mainly by Tregs and has a significant anti-inflammatory effect. It is widely involved in the immune response process *in vivo* and is closely related to the occurrence and development of inflammatory diseases such as RA. Furthermore, it can induce Tregs to produce iTR35 cells with stronger anti-inflammatory function and a more stable effect. At the same time, IL-35 can significantly inhibit the abnormal differentiation of Th17 cells to reduce the secretion of IL-17, and then effectively inhibits the development of inflammation. IL-35 can inhibit the proliferation of FLS, angiogenesis, and bone destruction caused by inflammation through JAK-STAT, Ang2-Tie2, and Wnt-β-catenin signaling pathways to relieve the symptoms of RA greatly. In clinical research, IL-35 shows that it plays an important role in the pathogenesis of RA, and it may be used as a potential target for the future treatment of RA with the further exploration of IL-35 function.

## Data Availability

The original contributions presented in the study are included in the article/Supplementary Material, further inquiries can be directed to the corresponding author.

## Author’s Note

All authors made substantial contributions to all of the following: 1) the conception and design of the study, or acquisition of data, or analysis and interpretation of data, 2) drafting the article or revising it critically for important intellectual content, 3) final approval of the version to be submitted.

## Abbreviation

RA, Rheumatoid arthritis; IL-35, Interleukin-35; IL-12, Interleukin-12; Treg, T regulatory cells; G-CSF, Granulocyte colony-stimulating factor; EBI3, Epstein-Barr virus-induced gene three; gp130, glycoprotein130; iTr35, IL-35 induced Treg; Foxp3, Forkhead Box p3; TGF-β, Transforming growth factor-β; FLS, Fibroblast-like synoviocytes; MMP, Matrix metalloproteinase; CIA mouse, Collagen-induced model of rheumatoid arthritis mouse; VEGF, Vascular endothelial growth factor; Ang, Angiopoietin; HUVECs, Human umbilical endothelial cells; RANKL-RANK, Receptor activator of NF-κB ligand/receptor activator of NF-ΚB; JAK-STAT, Janus Kinase-signal transducer and activator of transcription; Tie2, TEK Tyrosine kinase, Endothelial; Dkk-1, Dickkopf-1; DAS28-ESR, DAS28 erythrocyte sedimentation rate; Th17, T Helper cell; NF-κB, Nuclear factor kappa-B; OPG, Osteoclastogenesis inhibitory factor; IFN-γ, Interferon-γ.
